# Supplementary material for: The Mis6 inner kinetochore subcomplex maintains CENP-A nucleosomes against centromeric non-coding transcription during mitosis
Source: Commun Biol. 2022 Aug 15;5:818. doi: 10.1038/s42003-022-03786-y (PMC9378642; doi:10.1038/s42003-022-03786-y)
Supplement: Supplementary file 1 — Supplementary Information [file 42003_2022_3786_MOESM1_ESM.pdf]

Supplementary Information

The Mis6 inner kinetochore subcomplex maintains CENP-A nucleosomes against centromeric non-coding transcription during mitosis

Hayato Hirai, Yuki Shogaki and Masamitsu Sato

Supplementary table 1

Supplementary figures 1~14

Supplementary table 1. *S. pombe* strains used in this study

Strain No.	Genotype	Figures
HH609	h^+ *cdc10-27 C::Padh21-cnp1-GFP-kan cnp3-tdtomato-hph sid4-CFP-nat leu1*	1a, b, S1a
HH614	h^{90} *cdc25-22 C::Padh21-cnp1-GFP-kan cnp3-tdtomato-hph sid4-CFP-nat*	1c, d, S1a, b
HH1521	h^- *alp12-1828 C::Padh21-cnp1-GFP-kan plo1-2mCherry-hph sid4-CFP-nat leu1 ura4 ade6-M216*	1e, f, S1a, b
SP1769	h^- *GFP-cnp1-nat leu1 ura4 lys1*	2a
HH1812	h^+ *GFP-cnp1-nat mis6-302-HA-bsd leu1 ura4 ade6-M216*	2a
HH1928	h^+ *cut9-665 GFP-cnp1-nat leu1 ade6-M216*	2a
HH1930	h^+ *cut9-665 GFP-cnp1-nat mis6-302-HA-bsd leu1 ura4 ade6-M216*	2a
HH1888	h^+ *GFP-cnp1-nat ndc80-mCherry-bsd leu1 ura4 ade6-M216*	2c, d
HH1890	h^+ *scm3*^{S55P}*-13myc-hph GFP-cnp1-nat ndc80-mCherry-bsd leu1 ura4 ade6-M216*	2c, d
HH1897	h^- *mis6-302 GFP-cnp1-nat ndc80-mCherry-bsd leu1 ura4 ade6-M216*	2c, d
HH1687	h^+ *alp12-1828 CO2::nmt1-GFP-L-cnp1-kan plo1-2mCherry-hph sid4-CFP-nat leu1 ura4 ade6-M210*	2f-j, 4d, e 5b, e, f, S4, S5 S7, S11, S12 S13, S14
HH1693	h^- *alp12-1828 CO2::nmt1-GFP-L-cnp1-kan plo1-2mCherry-hph sid4-CFP-nat mis6-302-HA-bsd leu1 ura4 ade6-M216*	2f, g 4d, e, 5e, f S4, S5a, S11a-c S12, S13, S14c
SG1487	h^- *alp12-1828 CO2::nmt1-GFP-L-cnp1-kan plo1-2mCherry-hph sid4-CFP-nat mis15-68-HA-bsd leu1 ura4 ade6-M210*	2h, S5b, S7c, S11d-f
SG653	h^- *alp12-1828 CO2::nmt1-GFP-L-cnp1-kan plo1-2mCherry-hph sid4-CFP-nat mis12-537-HA-bsd leu1 ura4 ade6-M216*	2i, S5c, S7a
SG442	h^{90} *alp12-1828 CO2::nmt1-GFP-L-cnp1-kan plo1-2mCherry-hph sid4-CFP-nat nuf2-2::ura4*$^+$ *leu1 ura4 ade6-M210*	2j, S5d, S7b
HH503	h^+ *mis6-GFP-kan Z2-CFP-atb2-nat leu1 ura4 his2 ade6-M216* +pREP1-*kis1-GBP-mCherry*	3b
HH1678	h^- *mis6-GFP-kan CO2::nmt1-mCherry-L-cnp1-hph Z2-CFP-atb2-nat leu1 ura4 ade6-M216* +pREP1-*kis1-GBP*	3c, d, S8a, b
HH1684	h^+ *mis6-GFP-kan CO2::nmt1-mCherry-L-cnp1-hph Z2-CFP-atb2-nat leu1 ura4 his2 ade6-M216* +pREP1	3c, d, S8a, b
HH333	h^- *leu1 ura4 ade6-M216* +pREP1	3e, S8c
HH337	h^- *leu1 ura4 ade6-M216* +pREP1-*kis1-GBP-mCherry*	3e, S8c
HH451	h^- *mis6-GFP-kan leu1 ura4 ade6-M216* +pREP1-*kis1-GBP-mCh*	3e, S8c
HH1607	h^- *mis6-GFP-kan leu1 ura4 ade6-M216* +pREP1	3e, S8c
FY336	h^- *cnt1::ura4*$^+$ *leu1 ura4-DS/E ade6-M210*	4a, S9
HH2136	h^- *mis6-302 cnt1::ura4*$^+$ *leu1 ura4-DS/E ade6-M210*	4a, S9
JY741	h^- *leu1 ura4 ade6-M216*	4b, 5d, S10
KA2130	h^{90} *mis6-302 leu1 ura4 ade6-M216*	4b, S10b
HH2001	h^- *alp12-1828 CO2::nmt1-GFP-L-cnp1-kan plo1-2mCherry-hph sid4-CFP-nat*	5b, S14a, b

	pob3::bsd leu1 ura4 ade6-M216	
HH2010	h^+ alp12-1828 CO2::nmt1-GFP-L-cnp1-kan plo1-2mCherry-hph sid4-CFP-nat spt6::$ura4^+$ leu1 ura4 ade6-M210	5b, S14a, b
HH2029	h^+ alp12-1828 CO2::nmt1-GFP-L-cnp1-kan plo1-2mCherry-hph sid4-CFP-nat pob3::bsd spt6::$ura4^+$ leu1 ura4 ade6-M210	5b, S14a, b
HH2061	h^{90} cut9-665 cen2<<lac0-kan-ura4+ his7+<<(dis1pro)-GFP-lacI spt6::$ura4^+$ leu1 ura4 his2 ade6-M216	5c
HH2066	h^{90} cut9-665 cen2<<lac0-kan-ura4+ his7+<<(dis1pro)-GFP-lacI leu1 ura4 his2 ade6-M216	5c
HH1971	h^- mis6-302 spt6-GFP-kan leu1 ura4 ade6-M210	5d
HH1975	h^- spt6-GFP-kan leu1 ade6-M216	5d
HH2009	h^- alp12-1828 CO2::nmt1-GFP-L-cnp1-kan plo1-2mCherry-hph sid4-CFP-nat spt6::$ura4^+$ leu1 ura4 ade6-M216	5e, f, S14c
HH2055	h^{+or-} alp12-1828 CO2::nmt1-GFP-L-cnp1-kan plo1-2mCherry-hph sid4-CFP-nat mis6-302-HA-bsd spt6::$ura4^+$ leu1 ura4 ade6-M216	5e, f, S14c
SG1432	$h^{+\ or\ -}$ cdc10-27 cnp3-tdtomato-hph sid4-CFP-nat leu1 ura4 ade6-M210	S1b
SG1428	$h^{+\ or\ -}$ cdc10-27 C::Padh21-cnp1-GFP-kan cnp3-tdtomato-hph sid4-CFP-nat leu1 ura4 ade6-M210	S1b
SG1410	$h^{-\ or\ 90}$ cdc25-22 cnp3-tdtomato-hph sid4-CFP-nat	S1b
SG1156	h^- alp12-1828 plo1-2mCherry-hph sid4-CFP-nat leu1 ura4 ade6-M216	S1b
HH1218	h^+ leu1 ura4 his2 ade6-M216	S2
SG1458	$h^{+\ or\ -}$ cut9-665 leu1 ura4 ade6-M216	S2
HH1885	h^{90} cnp1-1-HA-bsd leu1 ura4 his2 ade6-M216	S2
SG1497	h^- cnp1-1-HA-bsd cut9-665 leu1 ura4 ade6-M216	S2
HH322	h^- mis6-GFP-kan leu1 ura4 ade6-M216	S3
HH1820	h^- mis6-GFP-kan $scm3^{S55P}$-13myc-hph leu1 ura4 ade6-M216	S3
KRY211	h^+ mis6-2GFP-kan cnp3-tdtomato-hph sid4-CFP-nat leu1 ura4 ade6-M216	S6
SG366	h^+ mis12-537 mis6-2GFP-kan cnp3-tdtomato-hph sid4-CFP-nat leu1 ura4 his2 ade6-M216	S6a
SG342	h^+ nuf2-1::$ura4^+$ mis6-2GFP-kan cnp3-tdtomato-hph sid4-CFP-nat leu1 ura4 his2 ade6-M216	S6a
SG344	h^+ nuf2-2::$ura4^+$ mis6-2GFP-kan cnp3-tdtomato-hph sid4-CFP-nat leu1 ura4 his2 ade6-M216	S6a
SG1414	h^+ mis15-68 mis6-2GFP-kan cnp3-tdtomato-hph sid4-CFP-nat leu1 ura4 his2 ade6-M216	S6b
SG1456	h^- mis15-68 cnt1::$ura4^+$ leu1 ura4-D18 or ura4-DS/E ade6-M216	S9a
SG571	h^- mis12-537 cnt1::$ura4^+$ leu1 ura4-D18 or ura4-DS/E ade6-M210	S9b
SG594	h^- nuf2-2::$ura4^-$ cnt1::$ura4^+$ leu1 ura4-D18 or ura4-DS/E ade6-M210	S9b
FY10461	h^- mis15-68 leu1 ura4	S10a
MT111	h^+ mis12-537 leu1 ura4 his2 ade6-M216	S10b
YK2330	h^- nuf2-1::$ura4^+$ leu1 ura4 ade6-M210	S10b
HH2000	h^- alp12-1828 CO2::nmt1-GFP-L-cnp1-kan plo1-2mCherry-hph sid4-CFP-nat mis6-302-HA-bsd fft3::$ura4^+$ leu1 ura4 ade6-M216	S11a-c

SG1575	*h^- alp12-1828 CO2::nmt1-GFP-L-cnp1-kan plo1-2mCherry-hph sid4-CFP-nat mis15-68-HA-bsd fft3::$ura4^+$ leu1 ura4 ade6-?*	S11d-f

SP1769 is a gift from Y. Takayama, from which HH1812, HH1888, HH1890, HH1897, HH1928, and HH1930 were created. Original strains used for creation of YK1316, YK2330, HH2061, HH2066, SG342, SG344, SG442 and SG594 were gifted from Y. Hiraoka and A. Yamamoto. The original strain used for creation of HH609, HH614, HH1521 and SG1428 were gifted from Y. Watanabe and T. Sakuno. Original strains used for creation of HH1521, HH1687, HH1693, HH1928, HH1930, HH2000, HH2009, HH2010, HH2055, HH2061, HH2066, SG442, SG653, SG1156, SG1458, SG1487, SG1497, and SG1575 were gifted from T. Toda. Original strains used for creation of KA2130, MT111, HH1693, HH1812, HH1897, HH1930, HH2000, HH2136, SG366, SG571 and SG653 were gifted from M. Yanagida. FY336, FY10461, and FY11198 strains were provided from NBRP (originated from M. Yanagida), from which HH1885, HH2136, SG571, SG594, SG1414, SG1456, SG1487, SG1497, and SG1575 were made. Other strains are our stock.

Supplementary figures

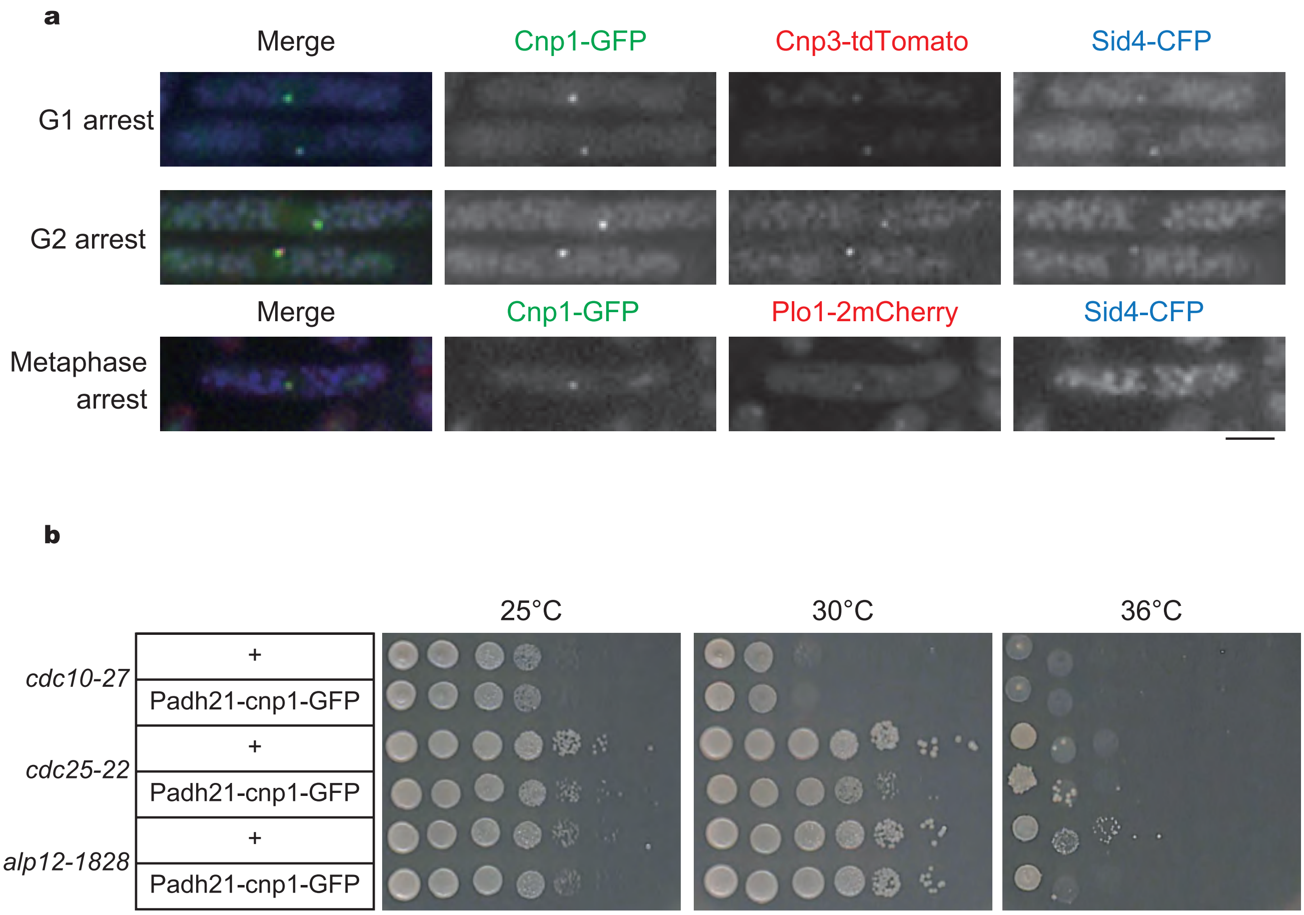

Supplementary Fig. 1 Strains expressing Cnp1-GFP under the *adh21* promoter show normal growth

(a) Cnp1-GFP expressed under the *adh21* promoter (Padh21-cnp1-GFP) localised at centromeres. Images showing Cnp1-GFP (green), Cnp3-tdTomato or Plo1-2mCherry (red; kinetochore or SPB marker), and Sid4-CFP (blue, SPB marker) in arrested G1, G2, and metaphase cells. Scale bar = 5 μm. **(b)** Spot assays indicate that introduction of Padh21-cnp1-GFP into cell cycle mutants (*cdc10-27*, *cdc25-22* and *alp12-1828*) did not cause growth defects. Ten-fold serial dilutions of cells were grown at 25°C, 30°C, and 36°C on YES agar plates.

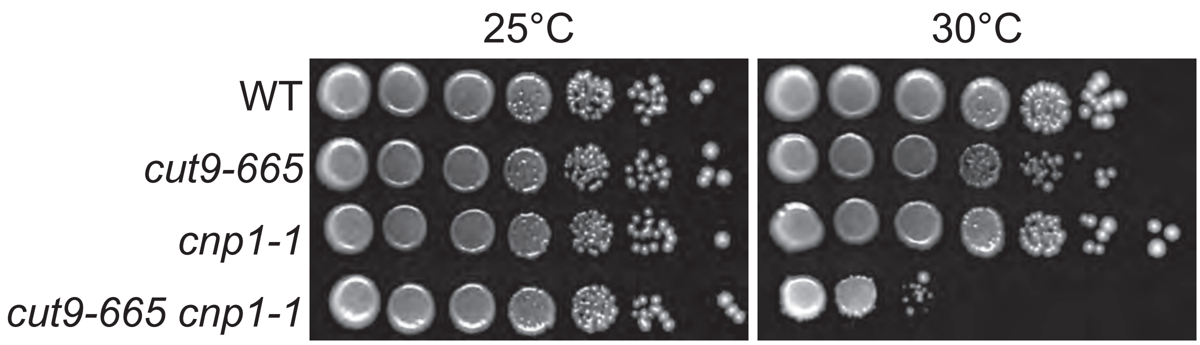

Supplementary Fig. 2 The *cut9-665 cnp1-1* double mutant exhibited server growth defects compared to each single mutant.

Ten-fold serial dilutions of cells were grown at 25°C and 30°C on YES agar plates.

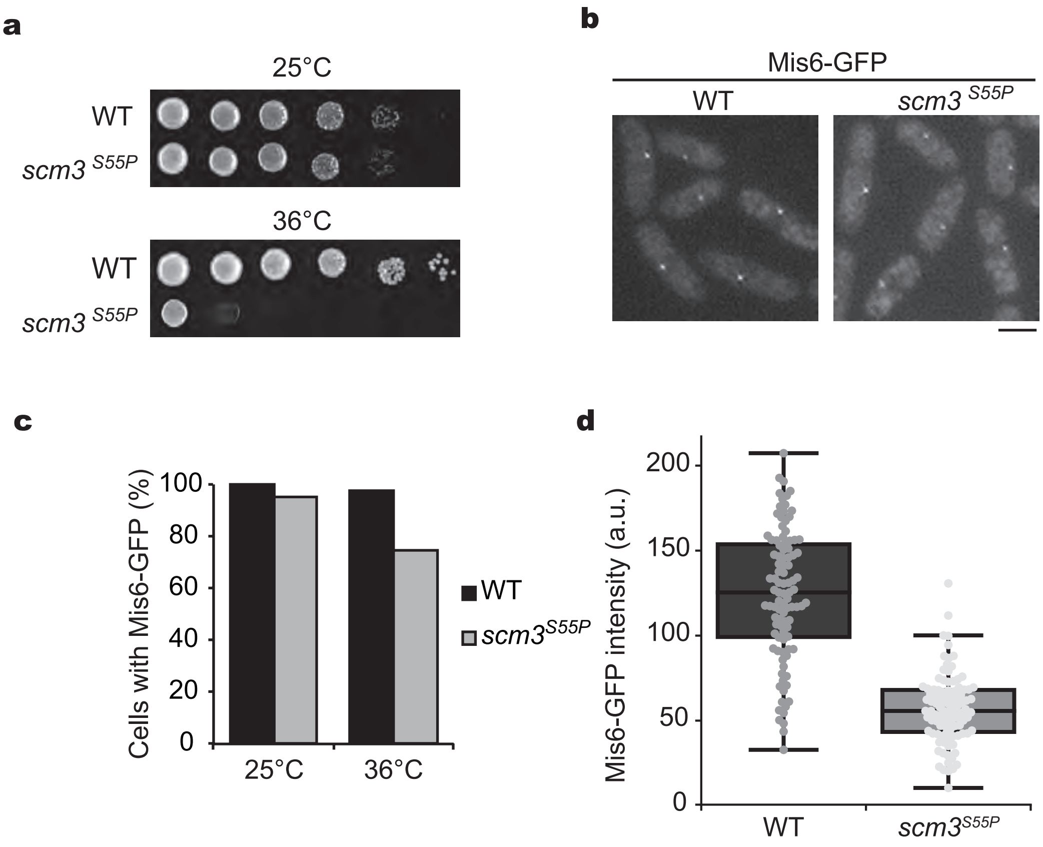

Supplementary Fig. 3 Centromeric localisation of Mis6 to centromeres in the *scm3*^{S55P} mutant
(a) Growth assays for indicated strains. Ten-fold serial dilutions of WT and *scm3*^{S55P} cells were grown at 25˚C or 36˚C. **(b)** Mis6-GFP localised to centromeres in *scm3*^{S55P} cells. Bar = 5 μm. **(c)** Percentages of cells with Mis6-GFP at centromeres in WT (black bars) and the *scm3*^{S55P} (grey bars) cells at 25°C and 36°C (6 h). WT (25°C), *n* = 157 cells; WT (36°C), *n* = 139 cells; *scm3*^{S55P} (25°C), *n* = 211 cells; *scm3*^{S55P} (36°C), *n* = 135 cells **(d)** Box plots showing the signal intensity of Mis6-GFP in WT (*n* = 99, black box) and the *scm3*^{S55P} (*n* = 99, grey box) cells at 36°C.

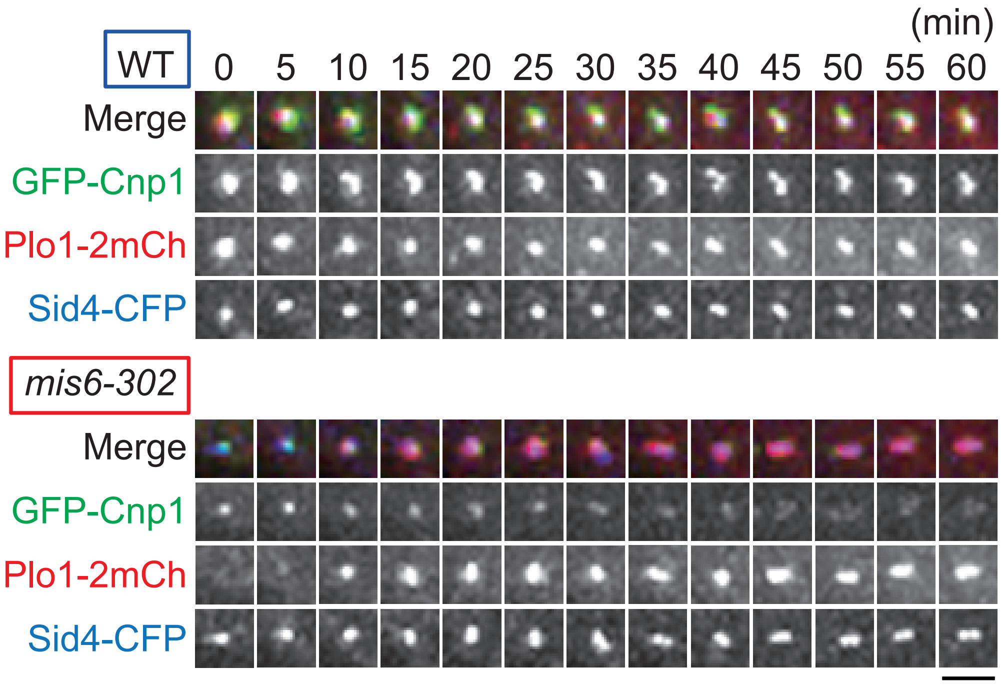

Supplementary Fig. 4 Mis6 is required for the maintenance of Cnp1 during metaphase

Time-lapse images showing GFP-Cnp1 signal (green) with Plo1-2mCherry (red) and Sid4-CFP (SPB) for 60 min during metaphase in WT and *mis6-302* cells. Bar = 2 μm.

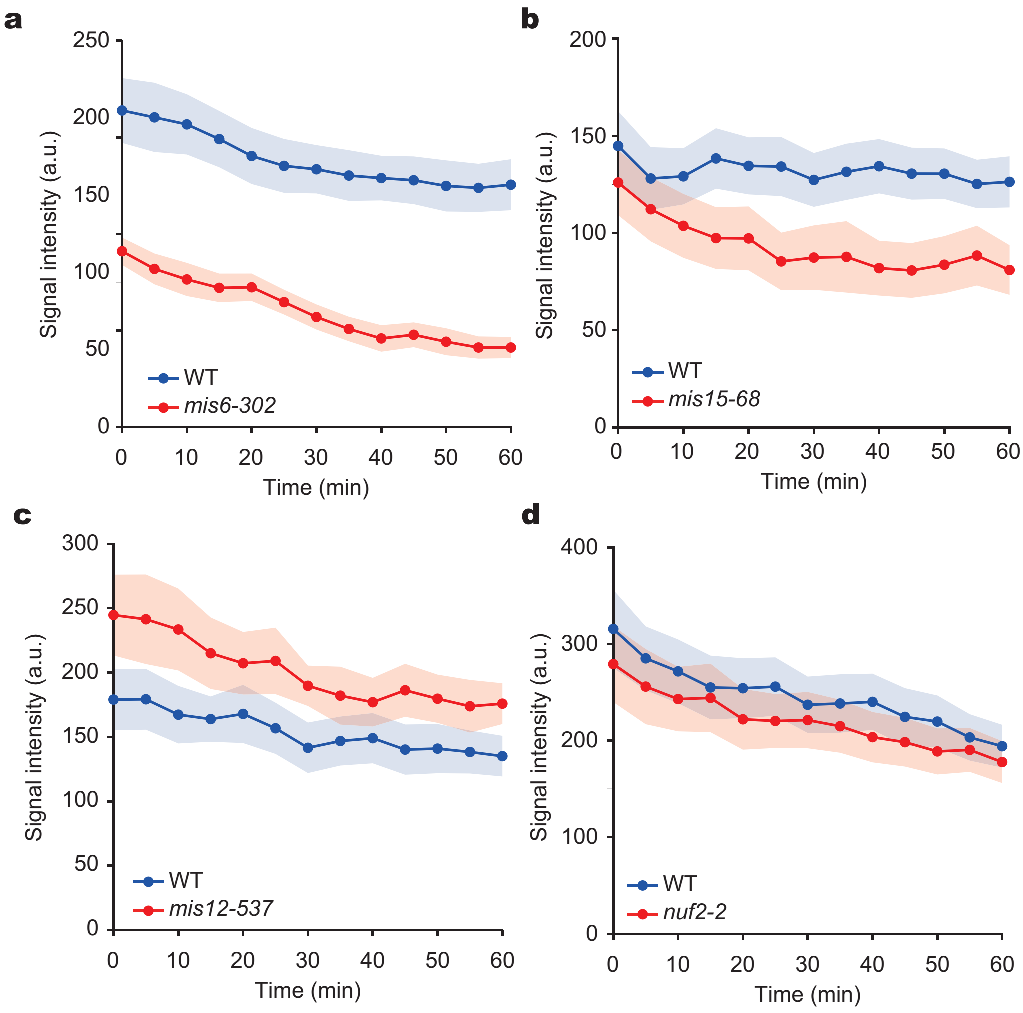

Supplementary Fig. 5 GFP-Cnp1 signal intensities without normalisation in each strain

Related to Fig. 2, the graphs show raw data for temporal kinetics of GFP-Cnp1 signal intensities during metaphase arrest in WT (blue solid lines) and each mutant strain (red solid lines). **(a)** WT, n = 16 cells; *mis6-302*, n = 20 cells. **(b)** WT, n = 20 cells; *mis15-68*, n = 12 cells. **(c)** WT, n = 11 cells; *mis12-537*, n = 6 cells. **(d)** WT, n = 21 cells; *nuf2-2*, n = 18 cells. Solid lines, means; coloured regions, standard errors (s.e.m.).

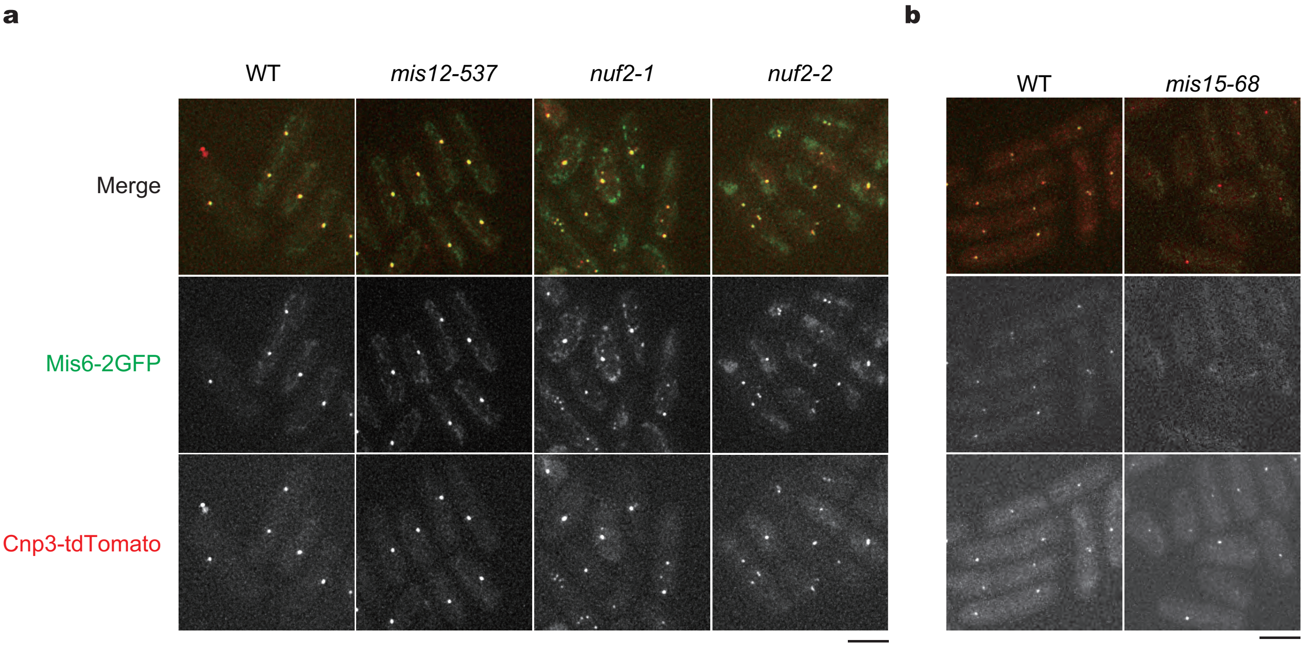

Supplementary Fig. 6 Localisation of Mis6 in the kinetochore mutants

Images showing Mis6-2GFP (green) with Cnp3-tdTomato (red, kinetochore). Mis6-2GFP localised normally in *mis12-537*, *nuf2-1* and *nuf2-2* cells as with WT cells (a), but not in *mis15-68* cells (b) at 36°C for 4 h. Bar = 5 μm.

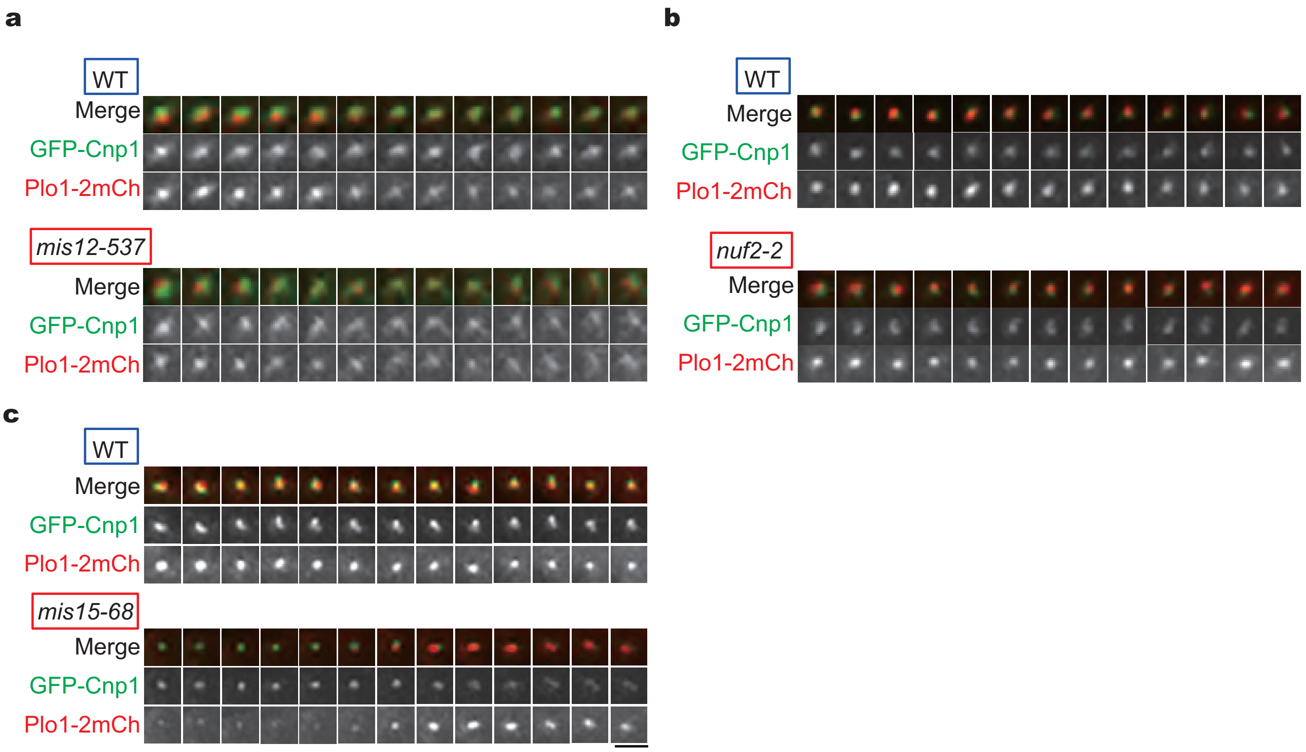

Supplementary Fig. 7 Cnp1 was maintained in *mis12-537* and *nuf2-2* cells, but not in *mis15-68* cells.

Images show temporal kinetics of GFP-Cnp1 signal with Plo1-2mCherry (red, SPB marker) in WT, *mis12-537* (a), *nuf2-2* (b), *mis15-68* cells (c). Bar = 2 μm.

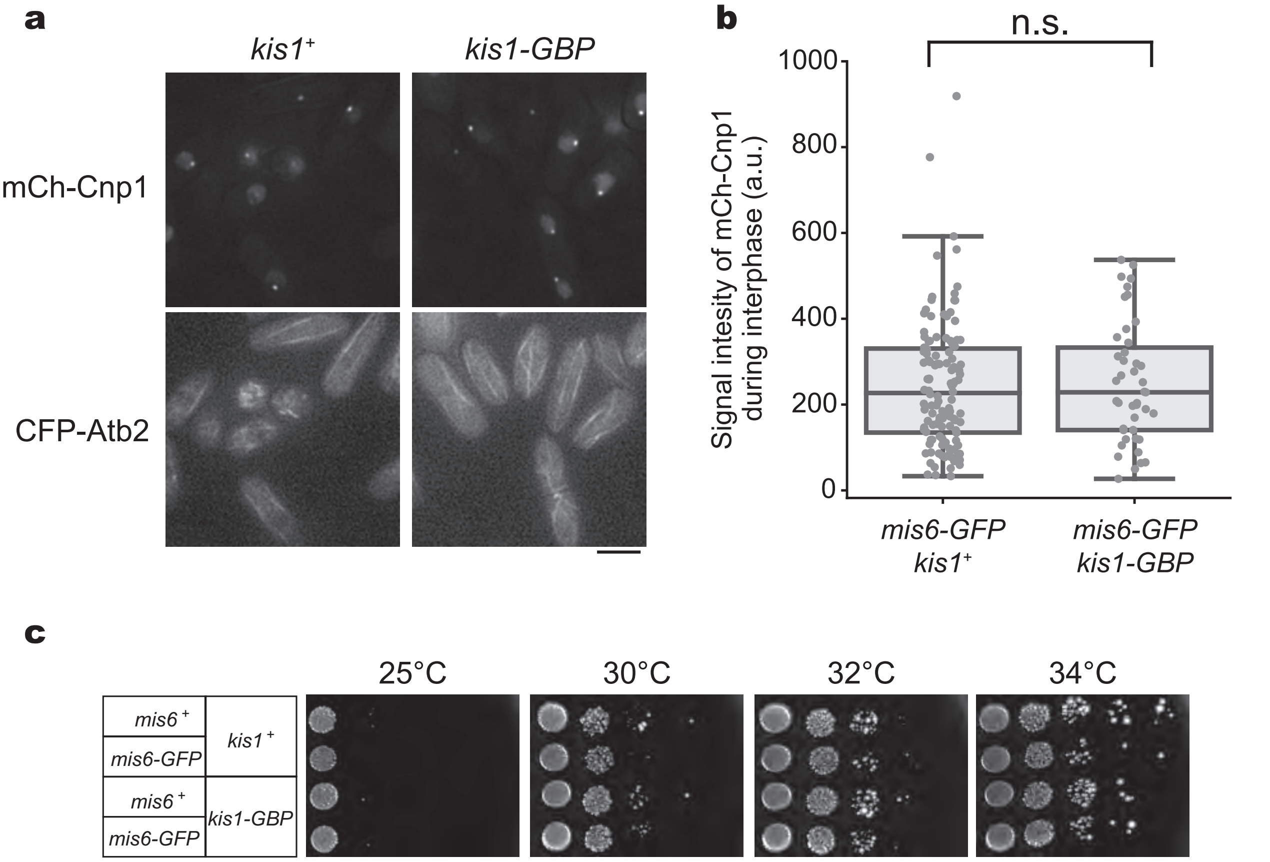

Supplementary Fig. 8 Localisation of Cnp1 in interphase is not affected in the *mis6-GFP kis1-GBP* strain

(a) mCherry-Cnp1 localised normally to centromeres in interphase cells of *kis1*+ *mis6-GFP* and *kis1-GBP mis6-GFP* strains. Bar = 5 μm. **(b)** Box plots showing fluorescence intensities of mCherry-Cnp1 at centromeres in interphase in the indicated strains. *kis1*+ *mis6-GFP*, n = 122 cells; *kis1-GBP mis6-GFP*, n = 43 cells. n.s., $p > 0.05$ (Welch's *t*-test). **(c)** Growth assays for indicated strains. Ten-fold serial dilutions of cells were grown in EMM at 25°C, 30°C, 32°C, and 34°C.

a

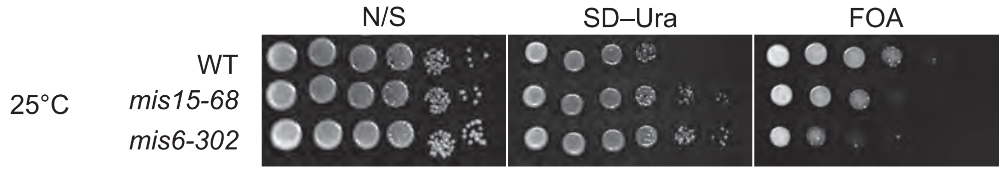

b

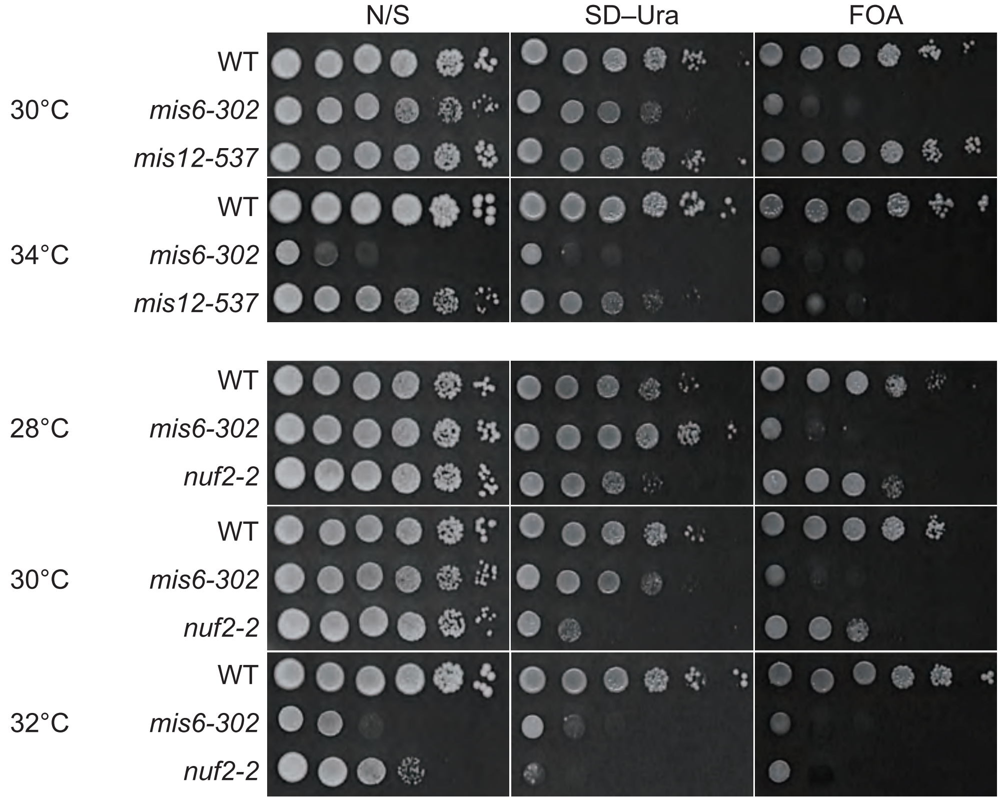

Supplementary Fig. 9 Silencing at the core centromere is intact in the *mis12* and *nuf2* but not *mis15* mutant cells

(a, b) Silencing assays. Ten-fold serial dilutions of WT, *mis15-68*, *mis6-302*, *mis12-537* and *nuf2-2* cells harbouring the *cnt1::ura4*$^{+}$ reporter gene were grown on the non-selective rich medium (N/S), SD–Ura as well as the FOA medium at indicated temperature.

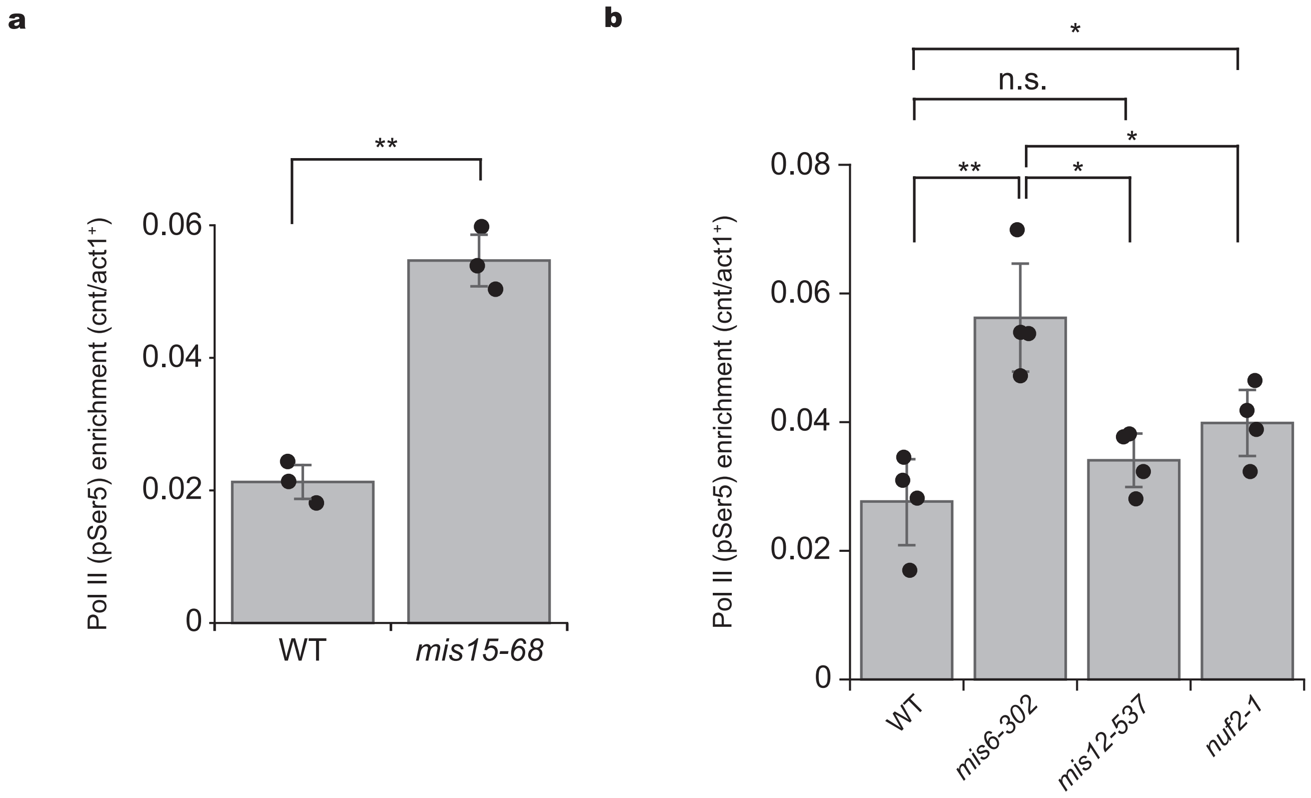

Supplementary Fig. 10 RNAPII was excessively accumulated to centromeres in *mis15* cells, but in neither of *mis12* nor *nuf2-1* cells

ChIP assays for RNAPII pSer5 in WT, *mis15-68*, *mis6-302*, *mis12-537* and *nuf2-1* cells at 36˚C (6 h in a, 4 h in b). The % input of the *cnt1* region was normalised to that of the $act1^{+}$ region. Error bars = s. d., $N = 3$ (a) and $N = 4$ (b) independent experiments. $p^{***} < 0.001$, $p^{**} < 0.01$, $p^{*} < 0.05$, n.s. $p > 0.05$ (Welch's *t*-test [two-tailed]). The data for WT and *mis6-302* in (b) shown in Fig. 4b are excerpt (reprise) of the entire data shown here.

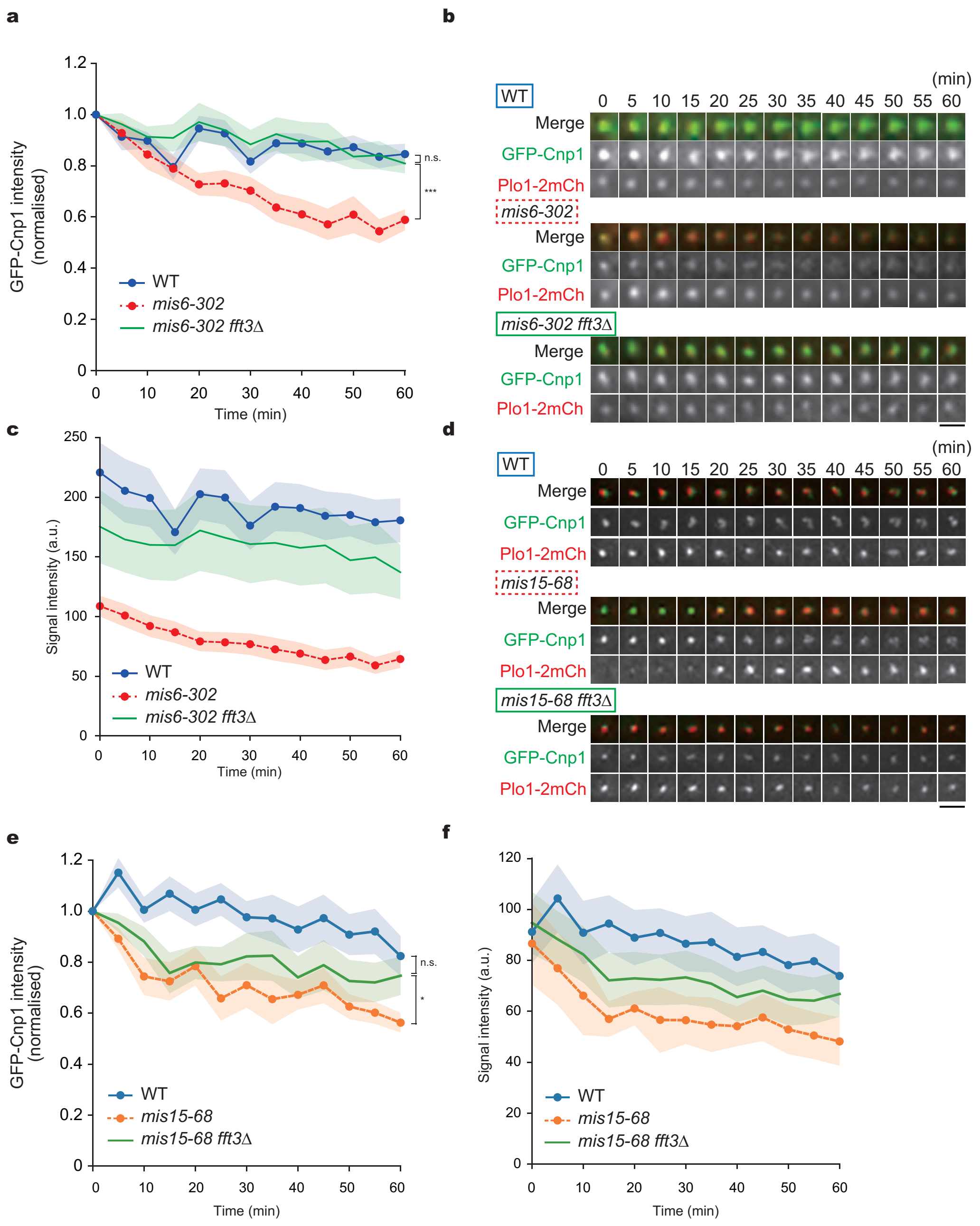

Supplementary Fig. 11 Assays for mitotic Cnp1 maintenance in *mis6-302 fft3*Δ and *mis15-68 fft3*Δ cells

(a, e) Temporal kinetics of the GFP-Cnp1 intensity in pro – metaphase of indicated cells. WT (blue solid line), n = 15 cells; *mis6-302* (red dashed line), n = 18; *mis6-302 fft3*Δ (green solid line), n = 13 (a). WT (blue solid line), n = 11; *mis15-68* (orange dashed line), n = 8; *mis15-68 fft3*Δ (green solid line), n = 12 (e). **(b, d)** Representative images of GFP-Cnp1 temporal kinetics are shown with Plo1-2mCherry (red, SPB). Bar = 2 μm. **(c, f)** Raw data without normalisation for (a) and (e). Means (solid and dashed lines) with error bars (s.e.m.; coloured regions) are shown. $p^{***} < 0.001$, $p^{*} < 0.05$, n.s. $p > 0.05$ (Welch's t-test [two-tailed]).

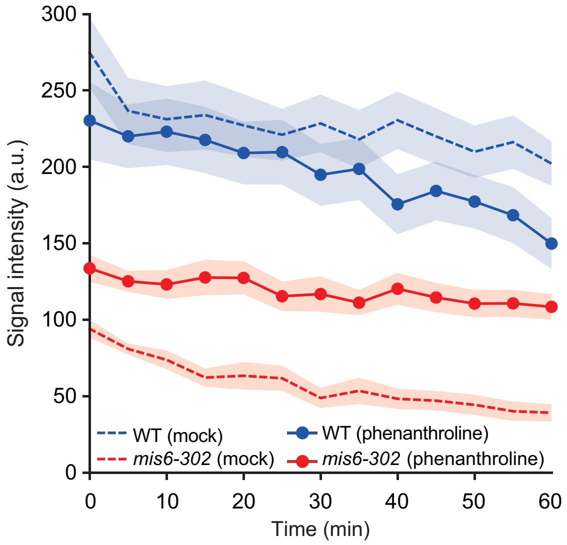

Supplementary Fig. 12 Raw data for Fig. 4e without normalisation

Raw data showing signal intensity of GFP-Cnp1 in indicated strains with or without an RNAPII inhibitor (1,10-phenanthroline). Means (solid and dashed lines) and error bars (s.e.m.; coloured regions) are shown.

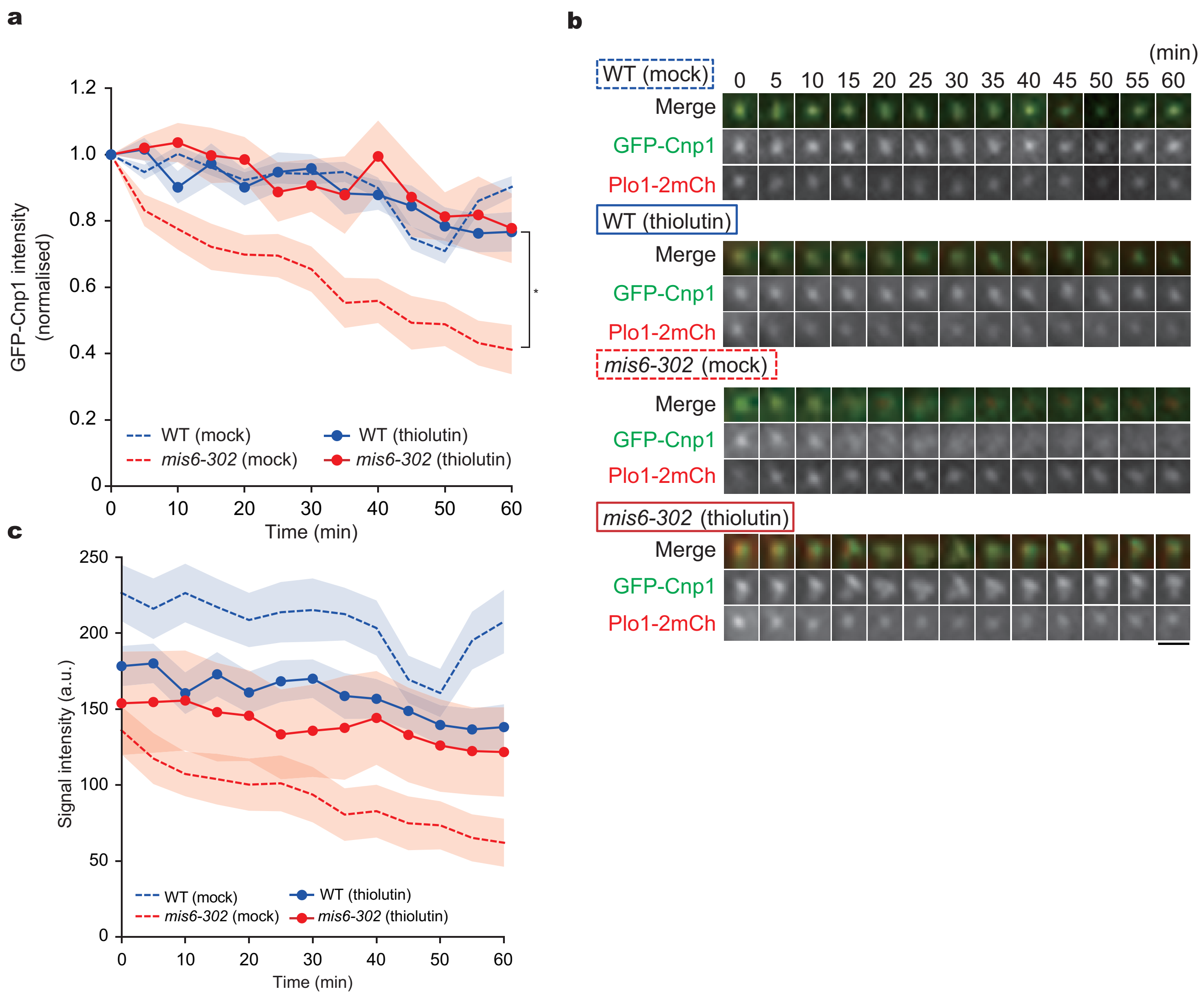

Supplementary Fig. 13 Another RNAPII inhibitor, thiolutin, also prevents dissociation of Cnp1

(a) Temporal kinetics of the GFP-Cnp1 intensity in pro – metaphase of WT and *mis6-302* cells in the presence of thiolutin. WT (mock, blue dashed line), $n = 23$ cells; *mis6-302* (mock, red dashed line), $n = 15$; WT (thiolutin, blue solid line), $n = 13$; *mis6-302* (thiolutin, red solid line), $n = 11$. The data have been normalised to intensities at 0 min. **(b)** Representative time-lapse images for cells expressing GFP-Cnp1 (green) and Plo1-2mCherry (red) in each genetic background. **(c)** Raw data for (a). Means (solid and dashed lines) and standard errors (s.e.m.; coloured regions) are shown. $p^* < 0.05$: Welch's *t*-test (two-tailed).

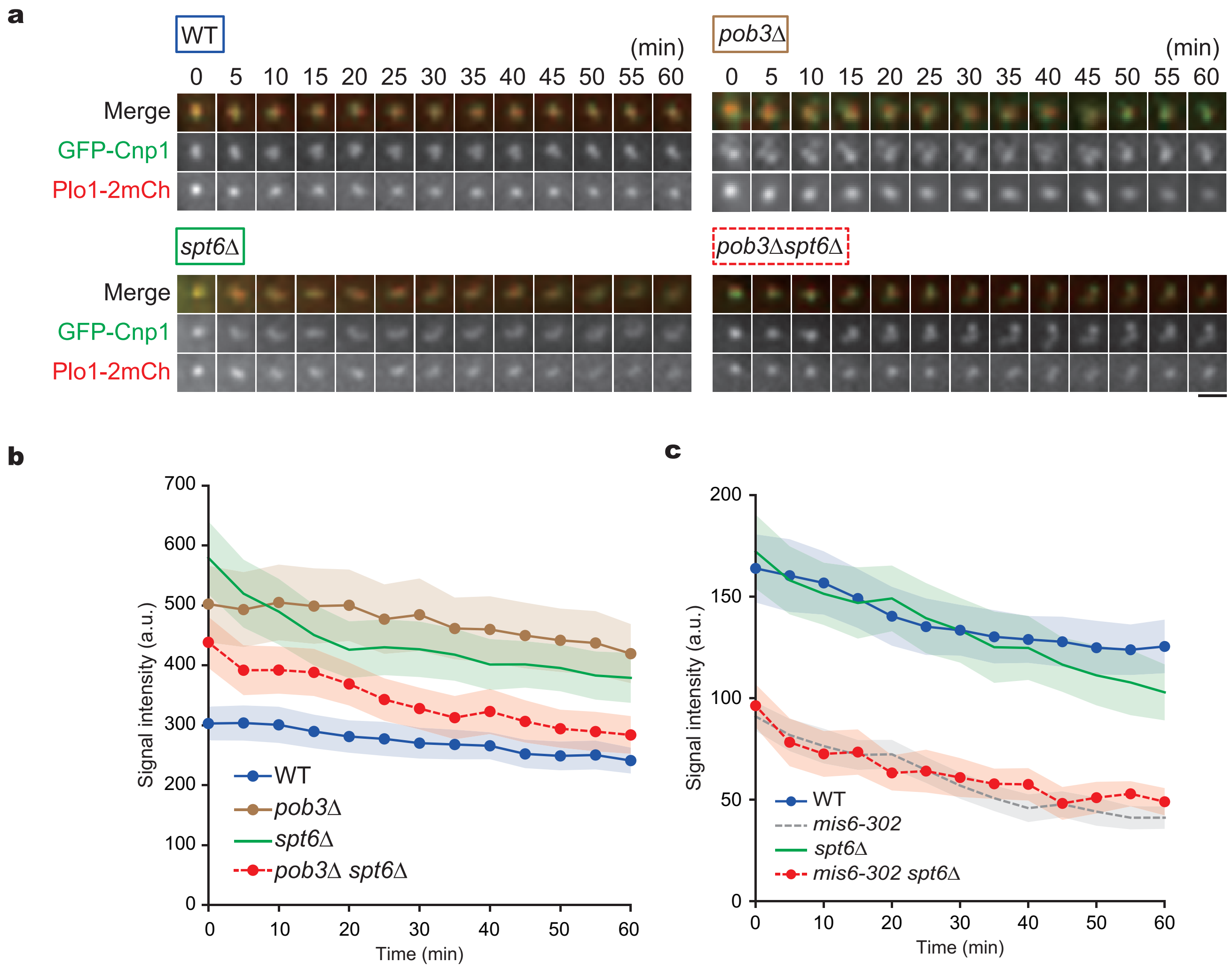

Supplementary Fig. 14 Assays for Cnp1 maintenance in FACT and Spt6 deficient cells

(a) Representative time-lapse images used for data presentation in Fig. 5b. Bar = 2 μm. **(b)** Raw data for Fig. 5b without normalisation. **(c)** Raw data for Fig. 5f without normalisation. Means (solid and dashed lines) and standard errors (s.e.m.; coloured regions).
