# Supplementary material for: The Mis6 inner kinetochore subcomplex maintains CENP-A nucleosomes against centromeric non-coding transcription during mitosis
Source: Commun Biol. 2022 Aug 15;5:818. doi: 10.1038/s42003-022-03786-y (PMC9378642; doi:10.1038/s42003-022-03786-y)
Supplement: Supplementary file 4 — Reporting Summary [file 42003_2022_3786_MOESM4_ESM.pdf]

nature portfolio

Corresponding author(s): MASAMITSU SATO

Last updated by author(s): Jul 14, 2022

Reporting Summary

Nature Portfolio wishes to improve the reproducibility of the work that we publish. This form provides structure for consistency and transparency in reporting. For further information on Nature Portfolio policies, see our Editorial Policies and the Editorial Policy Checklist.

Statistics

For all statistical analyses, confirm that the following items are present in the figure legend, table legend, main text, or Methods section.

n/a	Confirmed	
☐	☒	The exact sample size (n) for each experimental group/condition, given as a discrete number and unit of measurement
☐	☒	A statement on whether measurements were taken from distinct samples or whether the same sample was measured repeatedly
☐	☒	The statistical test(s) used AND whether they are one- or two-sided<br>*Only common tests should be described solely by name; describe more complex techniques in the Methods section.*
☒	☐	A description of all covariates tested
☒	☐	A description of any assumptions or corrections, such as tests of normality and adjustment for multiple comparisons
☒	☐	A full description of the statistical parameters including central tendency (e.g. means) or other basic estimates (e.g. regression coefficient) AND variation (e.g. standard deviation) or associated estimates of uncertainty (e.g. confidence intervals)
☐	☒	For null hypothesis testing, the test statistic (e.g. F, t, r) with confidence intervals, effect sizes, degrees of freedom and P value noted<br>*Give P values as exact values whenever suitable.*
☒	☐	For Bayesian analysis, information on the choice of priors and Markov chain Monte Carlo settings
☒	☐	For hierarchical and complex designs, identification of the appropriate level for tests and full reporting of outcomes
☒	☐	Estimates of effect sizes (e.g. Cohen's d, Pearson's r), indicating how they were calculated

Our web collection on statistics for biologists contains articles on many of the points above.

Software and code

Policy information about availability of computer code

Data collection	SoftWoRx v3.7.0 and v6.5.1 (Applied Precision) for image acquisition in microscopy. StepOne Real-Time PCR system (Applied Biosystems) for collecting ChIP data.
Data analysis	The Data Inspector command in SoftWoRx was used for measuring fluorescence intensity. Adjustment of images was used for Photoshop (ver.2021). Microsoft Excel and Python (3.9.0) was used for creating graphs and statistical processing.

For manuscripts utilizing custom algorithms or software that are central to the research but not yet described in published literature, software must be made available to editors and reviewers. We strongly encourage code deposition in a community repository (e.g. GitHub). See the Nature Portfolio guidelines for submitting code & software for further information.

Data

Policy information about availability of data

All manuscripts must include a data availability statement. This statement should provide the following information, where applicable:

- Accession codes, unique identifiers, or web links for publicly available datasets
- A description of any restrictions on data availability
- For clinical datasets or third party data, please ensure that the statement adheres to our policy

Plasmids created in this study are available in National BioResource Project (NBRP) in Japan (accession IDs: FYP5179, FYP5180, FYP5181, FYP5182). The data that support the findings of this study are available from the corresponding author upon reasonable request.

Field-specific reporting

Please select the one below that is the best fit for your research. If you are not sure, read the appropriate sections before making your selection.

☒ Life sciences ☐ Behavioural & social sciences ☐ Ecological, evolutionary & environmental sciences

For a reference copy of the document with all sections, see nature.com/documents/nr-reporting-summary-flat.pdf

Life sciences study design

All studies must disclose on these points even when the disclosure is negative.

Sample size	No sample-size calculation was performed before observation and measuring the signal intensity of fluorescences.<br>All sample-size was determined based on our previous fission yeast studies.
Data exclusions	No data were excluded from all analyses.
Replication	Fluorescence signal intensity measurements were performed on at least 6 cells and confirmed reproducible. For ChIP assay, at least three independent experiments were performed to confirm the reproducibility.
Randomization	Cells were randomly selected for microscopy observation and measurement of signal intensity, except for Supplementary Fig8c. In Supplementary Fig 8c, cells occasionally showed multiple mCherry-Cnp1 dots in the nucleus. We measured signal intensity of mCherry-Cnp1 that showed a single dot in the nucleus.
Blinding	n/a<br>Group allocation design is not commonly applied to fission yeast research

Reporting for specific materials, systems and methods

We require information from authors about some types of materials, experimental systems and methods used in many studies. Here, indicate whether each material, system or method listed is relevant to your study. If you are not sure if a list item applies to your research, read the appropriate section before selecting a response.

Materials & experimental systems

n/a	Involved in the study	
☐	☒	Antibodies
☒	☐	Eukaryotic cell lines
☒	☐	Palaeontology and archaeology
☒	☐	Animals and other organisms
☒	☐	Human research participants
☒	☐	Clinical data
☒	☐	Dual use research of concern

Methods

n/a	Involved in the study	
☒	☐	ChIP-seq
☒	☐	Flow cytometry
☒	☐	MRI-based neuroimaging

Antibodies

Antibodies used	For ChIP analysis, anti-RNA polymerase II (phosphoS5) polyclonal antibody (1:100; ab5131) and anti-GFP polyclonal anitbody (1:250; Clontech, 632592) were used.
Validation	In ChIP experiments, the validity of antibodies was confirmed by comparing with the negative control.
